# Editorial: Molecular Organization of Membranes: Where Biology Meets Biophysics

**DOI:** 10.3389/fcell.2017.00113

**Published:** 2017-12-13

**Authors:** Marek Cebecauer, David Holowka

**Affiliations:** ^1^Department of Biophysical Chemistry, J. Heyrovsky Institute of Physical Chemistry of the Czech Academy of Sciences, Prague, Czechia; ^2^Department of Chemistry and Chemical Biology, Cornell University, Ithaca, NY, United States

**Keywords:** nanodomains, tetraspanins, membrane properties, cell membrane, fluorescence microscopy, superresolution microscopy, membrane trafficking, caveolae

Membranes delimit the shapes of cells and their internal compartments, form a passive barrier between interior and exterior, but function also as organizing platforms for cellular processes. These highly diverse structures are formed by a large number of lipid and protein species. It is now generally accepted that both lipids and proteins are heterogeneously distributed in cell membranes (Cebecauer et al., 2010; Holowka and Baird, 2015; Sezgin et al., 2017). Such non-homogeneous organization of membranes linked to cellular functions attracts attention of scientists from fields as diverse as physiology, cell biology and biophysics. Indeed, the presence of various nanodomains in membranes forms a unifying link between the articles of this Research Topic.

The Research Topic collects 11 articles from authors with a broad background and focuses mainly on three issues resonating in membrane biology and biophysics: (i) physical properties of membranes contributing to cell membrane organization in molecular assemblies and domains; (ii) emerging role of tetraspanins, an evolutionarily conserved superfamily of membrane structural proteins, as critical players in membrane organization and (iii) novel tools to study cell membranes.

Various models were suggested to describe molecular organization of membranes and their capacity to respond to various chemical and physical stimuli. Five most frequently discussed models are summarized in the work of de la Serna et al. which also reports on the original works supporting or stimulating these hypotheses. Models are assessed for their ability to deal with a highly complex nature of membranes. This complexity is comprehensively described in sections focused on membrane structure and properties. The authors conclude that it is difficult to sum all membrane properties in one universal model. Fujimoto and Parmryd emphasize the importance of membrane asymmetry, interleaflet coupling and pinning in formation of lipid domains and membrane organization. These aspects of plasma membrane were overlooked in the past. Interleaflet coupling and pinning were studied only in a handful of works using model systems. Further studies will help to adapt current models of membrane organization to this rather new perspective.

Existence of lipid domains was believed to depend on sphingolipids and cholesterol (Simons and Ikonen, 1997). Whereas both lipid species are essential for plasma membrane structure and function, heterogenous distributions of cholesterol in domains has not been observed in tested membranes (Frisz et al., 2013; Honigmann et al., 2014). On the contrary, segregation of sphingolipids and glycosphingolipids into distinct membrane domains was demonstrated using a broad spectrum of techniques, as summarized in the work of Kraft. Implications of sphingolipid clustering on plasma membrane organization in domains are discussed. In analogy to lipids, proteins can form supramolecular assemblies associated with membranes. For example, caveolins interact at the membrane to create caveolae, flask-shaped domains decorating the surface of cells. Caveolae are involved in cellular processes such as lipid homeostasis or mechanotransduction. Even though these domains are relatively stable and easy to visualize, there is currently no consensus regarding their biogenesis. Han et al. report on issues associated with studies of caveolae biogenesis and suggest more intense use of disease-associated mutants to better understand these important membrane structures.

Full assembly of caveolae can lead to endocytosis and downregulation of membrane molecules from the cellular surface. Lou et al. report on the reverse process, exocytosis, and its importance for signaling events on T cells. Their work summarizes current knowledge on the diversity of exosomes involved in delivery of a principal receptor (TCR) and its effectors to the plasma membrane of T cells. The idea is that spatial separation of signaling molecules in vesicles prevents uncontrolled activation in the absence of stimulus. The impact of physical separation on receptor function is also studied in the only original research article of this Research Topic. Using *in silico* simulations, Kerketta et al. demonstrate a potential regulatory role of differential nanodomain partitioning of the two receptors, ErbB2 and ErbB3. Heterodimerisation and phosphorylation of these receptors depends on their relative accumulation in nanodomains. Whether in vesicles or in nanodomains, both articles emphasize the importance of compartmentalization and physical segregation of signaling molecules and the competence of cellular membranes to facilitate such processes. These works also underline the dynamic character of molecular separation in domains or vesicles. After stimulation, membrane domains move laterally and exosomes vertically fuse with bulk membranes to set up a new distribution allowing acceleration or deceleration of signaling events.

The presence of individual molecules in nanoscopic structures such as membrane domains or vesicles poses a challenge for standard imaging techniques (Cebecauer et al., 2010; Owen et al., 2010). Currently developed super-resolution techniques overcome this problem and allow visualization of molecules in cells with nanoscopic precision. Even though these techniques are prevalently used for static imaging, dynamic events can be visualized at nanoscopic level as reported in the work of Sergé. How emerging techniques help to better understand membrane-associated processes at nanoscopic level is illustrated on studies investigating dynamic reorganization of focal adhesions, and immune and neuronal synapses. These techniques are still in their early years and many improvements are needed. Here, Mateos-Gil et al. provide detailed description how to investigate distribution of membrane molecules using efficient and non-invasive labeling and super-resolution imaging. By adapting metabolic labeling and click chemistry combined with dSTORM, they characterized the whole glycome on the surface of cultured cells at nanoscopic level (Letschert et al., 2014).

Super-resolution imaging and improved labeling procedures accelerate research focused on membrane organization in general but, with their commercial availability, detailed characterization of individual molecules is emerging in the literature. Tetraspanins were commonly studied by standard microscopy. More recently, new imaging techniques provided a more detailed insight into the tetraspanin web (Zuidscherwoude et al., 2015). Two articles in this Research Topic summarize accumulating evidence on critical roles of tetraspanins in broad spectrum of cellular processes taking place on membranes. Termini and Gillette focuses on crosstalk between tetraspanins and signaling receptors (e.g., EGFR) or integrins involved in cell adhesion. Tetraspanins were shown to modulate dimerization, clustering and endocytosis of receptors. This effect is controlled by post-translational modifications of tetraspanins (e.g., palmitoylation or glycosylation). Halova and Draber report on accumulating evidence of cooperation between tetraspanins and membrane adaptors of the TRAP family (e.g., LAT) in immune cells. Functions of these proteins are regulated by their lipid modification but also by their colocalization in membrane nanodomains. The importance of cholesterol for this phenomenon is noted but no direct data are available yet. This can be caused by difficulties associated with characterization of protein-lipid interactions in cells of higher eukaryotes.

Singh in his work offers an alternative approach for investigation of protein-lipid interactions. Due to their simpler lipid metabolism and large panel of available genetic and cell-biological tools, he argues that yeasts provide an elegant tool for such studies.

We hope that this Research Topic will stimulate further studies to confirm or revise presented opinions.

## Author contributions

Both authors equally contributed to the drafting and revision of the editorial and approved it for publication.

### Conflict of interest statement

The authors declare that the research was conducted in the absence of any commercial or financial relationships that could be construed as a potential conflict of interest.
